# Apoprotein E and Reverse Cholesterol Transport

**DOI:** 10.3390/ijms19113479

**Published:** 2018-11-06

**Authors:** Godfrey S. Getz, Catherine A. Reardon

**Affiliations:** 1 Department of Pathology, University of Chicago, Chicago, IL 60637, USA; getz@bsd.uchicago.edu; 2 Ben May Institute for Cancer Research, University of Chicago, Chicago, IL 60637, USA

**Keywords:** apoprotein E, macrophage, cholesterol efflux, reverse cholesterol transport, hemopoietic stem cell progenitor cells, mimetic peptides

## Abstract

Apoprotein E (apoE) is a multifunctional protein. Its best-characterized function is as a ligand for low-density lipoprotein (LDL) receptor family members to mediate the clearance of apoB-containing atherogenic lipoproteins. Among its other functions, apoE is involved in cholesterol efflux, especially from cholesterol-loaded macrophage foam cells and other atherosclerosis-relevant cells, and in reverse cholesterol transport. Reverse cholesterol transport is a mechanism by which excess cellular cholesterol is transported via lipoproteins in the plasma to the liver where it can be excreted from the body in the feces. This process is thought to have a role in the attenuation of atherosclerosis. This review summarizes studies on the role of apoE in cellular cholesterol efflux and reverse cholesterol transport and discusses the identification of apoE mimetic peptides that may promote these pathways.

## 1. Introduction

Apoprotein E (apoE) is a multifunctional protein [1]. It is widely expressed, though not in all cell types, e.g., apoE is not expressed in enterocytes. It serves as a ligand for several cell surface receptors, particularly those involved in the clearance of apoB-containing chylomicron and very low density lipoprotein (VLDL) remnants (IDL) from the plasma and thus reduces plasma lipid levels. Among its other functions is its involvement in cholesterol efflux, especially from the macrophage foam cells that accumulate in the atherosclerotic vessel walls, and reverse cholesterol transport. Reverse cholesterol transport is a component of the mechanism for achieving whole-body cholesterol homeostasis involving cholesterol absorption and endogenous synthesis on the one hand and cholesterol excretion on the other. The most widely used experimental model of atherosclerosis is the *Apoe*-deficient (*Apoe^−/−^*) mouse, first described by Maeda and colleagues [2] and Breslow and colleagues [3]. The loss of apoE-mediated atherogenic lipoprotein clearance and reverse cholesterol transport functions contribute to hyperlipidemia-induced atherosclerosis in this murine model [4].

The two cells whose expression of apoE is most important for atherogenesis are hepatocytes in the liver and macrophages in the artery wall. Most of the apoE secreted from hepatocytes is associated with VLDL particles, where it mediates the clearance of remnants of these and intestinal-derived lipoproteins generated by lipolysis. Synthesis of apoE by macrophages mediates efflux of cholesterol from the cell, as is discussed below, but apoE may also serve as a ligand for remnant clearance. This is indicated by experiments in which apoE-expressing bone marrow is transplanted into *Apoe^−/−^* recipients [5,6]. The apoE from transplanted bone marrow cells is able to rescue the hyperlipidemia as well as the atherosclerosis of the *Apoe^−/−^* recipients. The extent to which the bone marrow-derived apoE contributes to reverse cholesterol transport in these models is not clear. However, under conditions when apoE derived from macrophages did not reduce hyperlipidemia, the expression of apoE in these cells reduced at least early atherogenesis [7,8], consistent with the idea that macrophage-derived apoE has anti-atherogenic functions that are independent of the plasma clearance of apoB-containing lipoproteins. The remainder of this review is devoted to the efflux of cholesterol from cholesterol-loaded cells mediated by exogenous and cell-autonomous apoE and to apoE’s role in reverse cholesterol transport.

## 2. Cholesterol Efflux and Reverse Cholesterol Transport

**Figure 1:**
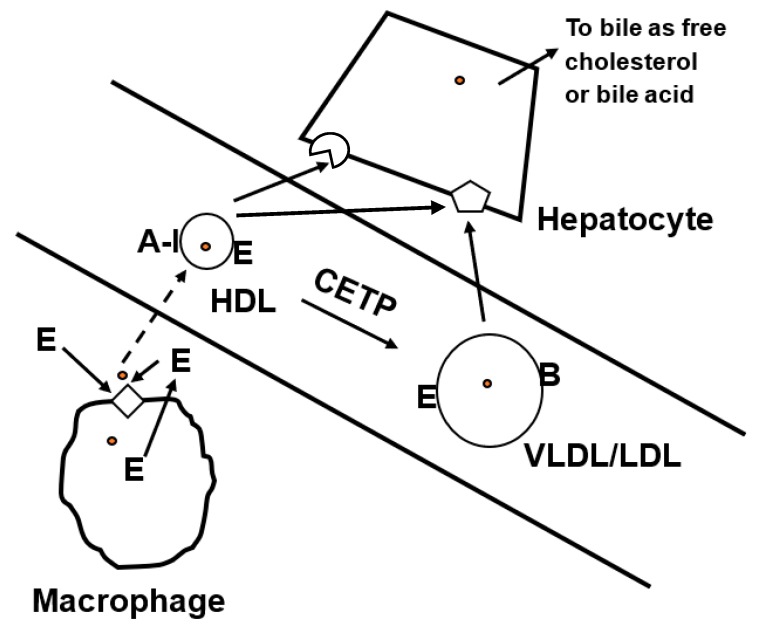
Apoprotein E (apoE) promotes cholesterol efflux and reverse cholesterol transport. Both exogenous and endogenous synthesized apoE can promote the efflux of cholesterol (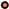) from cholesterol-loaded macrophages to form nascent discoidal high-density lipoprotein (HDL) after interaction with ABC transporters (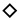). The nascent HDL with the effluxed cholesterol enters the plasma and via a number of steps (dashed line) is incorporated into mature spherical HDL particles. The HDL particles deliver the cholesterol to hepatocytes via interaction with scavenger receptor B1 (SR-BI) (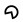) or low-density lipoprotein (LDL) receptor family members (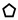). Alternatively, cholesteryl ester transfer protein (CETP) can mediate the transfer of cholesterol from HDL to VLDL and the effluxed cholesterol taken up by hepatocytes via the LDL receptor’s recognition of apoE or apoB.

Reverse cholesterol transport designates the process by which cholesterol from lipid-loaded peripheral cells, such as macrophage foam cells, passages through the plasma high-density lipoprotein (HDL) compartment to the liver and is excreted via the feces [9]. The first step in reverse cholesterol transport is the efflux of cholesterol from lipid-loaded peripheral cells. Macrophages take up oxidatively modified low-density protein (LDL) by scavenger receptor-mediated endocytosis. The lipoprotein-derived cholesterol released from the lysosome is esterified by acyl-coenzyme A:cholesterol acyltransferase and stored in the cytosol as cholesteryl ester-rich lipid droplets. The cholesteryl esters in the lipid droplets are hydrolyzed by neutral cholesteryl ester hydrolase in the cytosol or by lysosomal acid lipase following the transfer of the lipid droplet to lysosomes via autophagosomes to generate free cholesterol. The free cholesterol moves to the plasma membrane where it is available for efflux. Four pathways mediate the efflux of cholesterol: simple diffusion, facilitated diffusion mediated by scavenger receptor B1 (SR-BI), and efflux mediated by the transporters ATP binding cassette (ABC)A1 or ABCG1 in the presence of extracellular acceptors, such as lipid-poor apoproteins or more mature HDL, respectively [9,10,11]. The mechanism most studied is that mediated by the transporter ABCA1, the protein whose genetic deficiency gives rise to Tangier disease, characterized by excessive storage of cholesteryl ester in lymphoid tissues. The level of ABCA1 can be transcriptionally upregulated by activation of the liver X receptor, a nuclear receptor that is activated by oxysterol derivatives of cholesterol. Although lipid-poor apoA-I is the dominant acceptor of the cholesterol and phospholipoid effluxed from the outer leaflet of the plasma membrane enriched in free cholesterol by ABCA1, other proteins may function in this respect, including apoE [12] (Figure 1). The association of the effluxed lipids with apoproteins generates preβ-discoid HDL. This nascent HDL is acted upon by lecithin cholesterol acyl transferase to form cholesteryl ester containing the fatty acid derived from the 2-position of lecithin. This forms the core of HDL as it is transformed from a nascent discoid to a mature spherical particle. The HDL containing the effluxed cholesterol can be taken up by hepatocytes mediated by SR-BI or the LDL receptor family members. Alternatively, the cholesterol in the HDL can be transferred to apoB-containing lipoproteins via the action of cholesteryl ester transfer protein (CETP) and the effluxed cholesterol transferred to the liver by the uptake of these particles by LDL receptor. Once in the liver, the cholesterol can be excreted from the body in feces by its transfer to the bile either as free cholesterol or after conversion to bile acids.

It was long thought that this pathway involving hepatic biliary excretion was the sole pathway for cholesterol excretion. More recently, transintestinal cholesterol excretion (TICE) has been described [13]. This pathway does not require HDL as a mediator. Instead, hepatic cholesterol is packaged in apoB-containing lipoproteins, which are endocytosed by enterocytes on the basal lateral membrane by members of the LDL receptor family; the cholesterol released from the lysosomes is secreted into the intestinal lumen, mediated by ABCG5/ABCG8 and other transporters. It has been estimated that TICE is responsible for ~35% of cholesterol excretion [14].

Cholesterol efflux is usually measured in vitro using cultured macrophage cells loaded with cholesterol by incubating the cells with acetylated LDL (AcLDL), which is recognized by scavenger receptors, and radioactive free cholesterol. Efflux of cholesterol to acceptor molecules is assessed by the release of radioactivity from the loaded cells into the culture medium. A variety of macrophage models have been studied. These include the mouse macrophage J774A.1 and RAW 264.7 cells, the human macrophage THP-1 cells, mouse peritoneal or bone marrow-derived macrophages, and human monocyte-derived macrophages. While J774 and RAW cells are most frequently studied, these cells differ from the other cells in that they do not express endogenous apoE, and this likely contributes to the idea that apoE is not a major acceptor or promoter of effluxed cholesterol.

Reverse cholesterol transport is by definition studied in vivo. Two major methods are available for such experiments. The most widely used method is the macrophage to feces reverse cholesterol assay described by Rader and colleagues [15]. Here, J774 cells are loaded in culture with modified LDL in the presence of radioactive free cholesterol similar to the in vitro studies. As J774 cells express low levels of ABCA1, the cells are usually treated with a cAMP analogue to upregulate ABC transporters [16]. The labeled cells are injected into the peritoneal cavity of mice, following which radioactivity in the plasma is measured at various times after injection, and the fecal and liver radioactivity is assessed after 24 or 48 h. The J774 cells may be modified genetically to assess the contribution of targeted macrophage genes in the process, and the cells can be injected into mice with genetic modifications of proteins. Peritoneal macrophages or bone marrow-derived macrophages from genetically modified mice have been used as well. This has been a very valuable approach and has yielded important information about the regulation of reverse cholesterol transport and its implication in atherosclerosis and cardiovascular diseases [16]. However, a major limitation is that it relies on the transfer of radioactive cholesterol from the injected macrophages into the different tissue compartments. It does not assess changes in cholesterol mass or the flux of cholesterol from the plasma into the cells. Nor does it inform us about the possible contribution to the cholesterol pool by endogenous synthesis. To address some of these issues, Sontag and colleagues described a procedure in which macrophages loaded with cholesterol, with or without radiolabeled free cholesterol, are sequestered in alginate capsules under the skin [17]. The capsules are easily formed and recovered 24–48 h after injection. The sequestered macrophages are little changed in number or viability over the experimental period. Radioactivity in the plasma, liver, and feces provides a measure of the efflux and transport of cholesterol from macrophages to the feces, as does the Rader procedure. However, in addition, the mass and specific activity of the cholesterol in the recovered macrophages can be measured to provide a more comprehensive picture of cholesterol homeostasis in the macrophage. Comparison of the initial and final mass of free cholesterol and cholesteryl ester assesses the net flux of macrophage cholesterol. The initial and final specific activity of macrophage cholesterol is indicative of an influx of cholesterol from the plasma lipoprotein uptake and/or from endogenous cellular synthesis. Parenthetically, this procedure is also applicable to other cell types, such as lymphocytes. Both methods assess the flux of cholesterol from macrophages to the liver and feces, but the alginate encapsulation method provides additional information about cholesterol homeostasis in the macrophage.

Cholesterol content of thioglycolate-induced peritoneal macrophages or macrophages from other tissue sources from hyperlipidemic mice can be measured as well. However, this provides a much less direct assessment of cholesterol homeostasis since is not possible to compare the same cell pool across physiological time.

## 3. Cholesterol Efflux and ApoE

**Figure 2:**
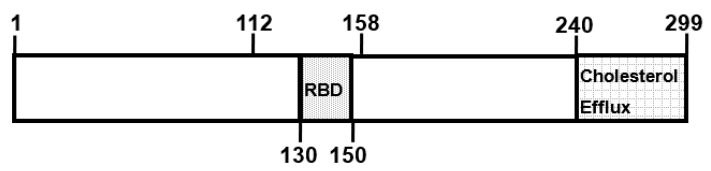
Domains of human apoE. The LDL receptor-binding domain (RBD) is located between residues 130 and 150 and the cholesterol efflux domain between residues 240 and 299. The apoE isoforms differ in the amino acids at positions 112 and 158. Both residues are arginines in apoE4, cysteines in apoE2, and cysteine at 112 and arginine at 158 in apoE3.

As mentioned, there are four pathways for the efflux of cholesterol from macrophages [10]. Efflux mediated by ABCA1 involves the transfer of cell membrane cholesterol and phospholipid to lipid-poor acceptors, of which apoA-I is the most studied. However, other amphipathic apoproteins, notably apoE, may also function as acceptors. This has been demonstrated with the human apoE isoforms apoE2, apoE3, and apoE4 incubated with ABCA1-expressing HeLa cells and J774 cells [18]. Structurally, apoE is organized as an N-terminal (residues 1–191) domain containing a four-helical bundle, and a C-terminal (residues 201–299) domain containing a series of amphipathic α-helices (Figure 2). The C-terminal domain of apoE3 (residues 222–299) essentially duplicates the efflux capacity of intact apoE3 [18]. The capacity of the C-terminal domain of apoE to promote cholesterol efflux was confirmed using chimeric molecules involving the interchange of apoA-I and apoE N- and C-terminal domains. The chimeric molecule consisting of the apoA-I N-terminal domain and the apoE C-terminal domain (apoA-1 (1–180)/apoE (201–299)) is more effective as a cholesterol acceptor from J774 cells than are the intact apoproteins or the reciprocal chimera [19]. As mentioned, J774 cells do not express apoE. However, these cells have been engineered to express human apoE3 under the control of the cytomegalovirus promoter [20]. This provides an opportunity to evaluate potential differences between the effect of endogenous and exogenous apoE on cholesterol efflux and cellular cholesterol homeostasis without the confounding effect of the sterol-mediated upregulation of apoE gene expression observed upon the cholesterol loading of macrophages [21]. Using these J774+E cells, Mazzone and colleagues demonstrated that endogenous apoE is more efficient in supporting sterol efflux than is exogenous apoE [22,23]. The endogenous expression of apoE results in the secreted apoE associating with the plasma membrane, where it binds to the cell surface LDL receptor, proteoglycans, and membrane lipids [24,25]. Reducing secreted apoE’s association with these cell surface molecules reduces cholesterol efflux. This cell surface association is not observed with exogenous apoE and, thus, the increased sterol efflux mediated by exogenous apoE on control J774 cells is not influenced by these manipulations. Interestingly, the increased efflux observed with J774+E cells is independent of ABCA1 [26]. These observations are not unique to the J774 cell line since similar observations are made with another murine line—RAW ± E [27]—and with mouse peritoneal macrophages from wild-type and *Apoe^−/−^* mice [28].

In the human macrophage THP-1 cells, cholesterol loading results in about a 15-fold increase in apoE mRNA [29]. As expected, apoE and the LDL receptor are reciprocally regulated by cholesterol loading. In contrast, human monocyte-derived macrophages do not show an upregulation of apoE expression upon loading with cholesterol, though these cells do efflux cholesterol upon loading, even without an exogenous sterol acceptor [30]. This efflux is nevertheless apoE-dependent, as it is abrogated by antibody-mediated removal of apoE.

There are three major human apoE isoforms. Relative to apoE3, which is the most prevalent isoform, apoE4 and apoE2 contain a single amino acid substitution at residues 112 and 158, respectively [31]. ApoE2 has a reduced ability to bind to the LDL receptor, while apoE4 has a slightly higher affinity compared to apoE3. There is no difference in the ability of exogenous human apoE isoforms to promote cholesterol efflux from cells [18]. However, using cholesterol-loaded monocyte-derived macrophages from individuals homozygous for the three isoforms, apoE2 was shown to promote more cholesterol efflux than apoE3 and apoE4 [32].

The interaction of secreted apoE with the cell surface LDL receptor is illustrated using peritoneal macrophages derived from *ApoE* gene replacement mice expressing the human apoE isoforms in place of the murine apoE gene [33]. When the LDL receptor is upregulated by treatment with simvastatin, both apoE secretion into the medium and sterol efflux are modified depending upon the capacity of the apoE isoform to interact with the receptor. Thus, increased LDL receptor expression reduces apoE4 secretion and cholesterol efflux, while apoE2 secretion and sterol efflux are not affected. These differences in cholesterol efflux mediated by the apoE isoforms reflect the differences in the binding affinity of isoforms to the receptor. Consistent with this, increasing the expression of the LDL receptor in apoE4 gene replacement mice decreases cholesterol efflux from peritoneal macrophages, and the transplantation of bone marrow from these mice into *Ldlr^−/−^* mice leads to enhanced atherosclerosis [34]. These in vitro and in vivo effects of increased expression of the LDL receptor are not observed in apoE3 gene replacement mice.

ApoE influences reverse cholesterol transport beyond the macrophage cell surface. For example, apoE secreted by macrophages may associate with human HDL_2_, which, in the absence of CETP, is able to expand to carry a larger cholesterol load to the liver for cholesterol secretion into the bile [35]. Removal of apoE from this HDL prevents its expansion.

The acceptor quality of various plasma or serum fractions has been evaluated using the plasma of genetically distinct strains of mice. Serum HDL was prepared by depletion of apoB-containing lipoproteins [12]. The Heinecke laboratory evaluated cholesterol efflux using J774 cells stimulated with cAMP and BHK cells with inducible ABCA1 expression. Serum HDL from *Apoa1^−/−^* and *Apoe^−/−^* mice has poor cholesterol efflux capacity from macrophages, and serum HDL from mice lacking both proteins has very little efflux capacity [36]. However, ABCA1-specific efflux is primarily determined by apoA-I, as serum HDL isolated from *Apoe^−/−^* mice has similar efflux capacity compared to wild-type serum HDL. Similar analyses were reported using serum HDL from five different inbred strains of mice [37]. These strains exhibit differences in HDL size and apoprotein content. The surprising finding is that apoE levels on HDL are negatively correlated with macrophage cholesterol efflux capacity. Indeed, when normalized for HDL particle concentration, *Apoe^−/−^* serum HDL is a better cholesterol acceptor than is wild-type serum HDL. These studies suggest that apoE on HDL may be a negative regulator of cholesterol efflux.

## 4. Reverse Cholesterol Transport and ApoE

Most of the above data reflect experiments that narrowly focus on apoE and cholesterol efflux from cultured macrophages. Few studies have examined the impact of macrophage and systemic apoE on reverse cholesterol transport in vivo. In one study, macrophage to feces reverse cholesterol transport was compared between wild-type (apoE-expressing) mice injected with cholesterol loaded wild-type peritoneal macrophages and *Apoe^−/−^* mice injected with cholesterol-loaded *Apoe^−/−^* peritoneal macrophages [28]. A reduction in reverse cholesterol transport was observed in the *Apoe^−/−^* mice, but the *Apoe^−/−^* mice have higher plasma lipid levels and lower HDL levels than the wild-type mice, confounding the interpretation. On the other hand, using wild-type mice as the recipients so that there was no difference in plasma lipids and lipoproteins, again the mice receiving *Apoe^−/−^* macrophages exhibited reduced reverse cholesterol transport compared to mice receiving wild-type macrophages. A role for systemic apoE’s impact on reverse cholesterol transport was also ruled out by the injection of wild-type cholesterol-loaded macrophages into wild-type and *Apoe^−/−^* recipients. These studies demonstrate that macrophage apoE can contribute to reverse cholesterol transport in vivo. This conclusion was obtained using injected peritoneal macrophages and whether this is also true of macrophages in the artery wall is unknown.

Recent in vivo experiments suggest that apoE may network with other cell functions to influence macrophage cholesterol homeostasis. Becker and collaborators described a “macrophage sterol responsive network” in which a number of proteins were shown to be either up- or downregulated in cholesterol-loaded peritoneal macrophages isolated from Western diet-fed *Ldlr^−/−^* mice [38]. These cells accumulate cholesterol and its esters but reveal a substantial downregulation of apoE protein expression, in contrast to what has been reported for macrophages incubated with AcLDL in culture [21,29]. The basis for these different in vitro culture and in vivo phenotypes with respect to apoE expression is not clear. Reardon, Becker, and colleagues have extended their studies to macrophages in obese/insulin-resistant mice in a search for mechanisms that might account for the increased risk of atherosclerosis in type 2 diabetes [39]. The induction of insulin resistance in *Ldlr^−/−^* mice fed a Western-type diet revealed the significance of IFNγ in mediating an increase in peritoneal macrophage cholesterol accumulation, a decrease in macrophage apoE secretion, and increased atherosclerosis. This was not observed in hyperlipidemic *Ldlr^−/−^* mice fed a low-fat, high-cholesterol diet that did not induce insulin resistance. The impact of IFNγ on the macrophage apoE phenotype was also applicable to other macrophages, including murine arterial macrophages. The effect of reduced macrophage apoE secretion on reverse cholesterol transport has not yet been demonstrated in this model.

## 5. Cholesterol Efflux, Hematopoiesis, and ApoE

The monocyte-derived macrophage is a central cell in atherogenesis [40], and increased numbers of monocytes in the blood are associated with increased influx of monocytes into atherosclerotic plaques [41]. Cholesterol homeostasis is an important regulator of leukocytosis, affecting both neutrophils and monocytes. Seminal studies showed that hemopoietic stem cell progenitor cells (HSPCs) that give rise to these innate immune cells proliferate more rapidly when their cholesterol content increases as a result of the deficiencies of the transporters ABCA1 and ABCG1 and decreased cholesterol efflux [42]. Leukocytosis is observed in high-fat, high-cholesterol diet-fed *Apoe^−/−^* mice, along with increased levels of HSPCs and GM-CSF [43,44]. Competitive bone marrow transplantation studies demonstrated that HSPCs from *Apoe^−/−^* mice proliferate more rapidly, suggesting that the synthesis of apoE by HSPCs suppresses proliferation. ApoE was found to be associated with the surface of the HSPC in complex with cell surface proteoglycans, and removal of this surface pool of apoE promotes cell proliferation. Since non-cell-associated apoE is ineffective in restoring the HSPC levels to baseline levels, it has been suggested that cell-autonomous apoE associated with proteoglycans collaborates with the two ABC transporters to remove the excess cholesterol from the HSPC to suppress proliferation. An effect of apoE on monocytosis was also observed using hypomorphic *Apoe^h/h^ Ldlr^−/−^* mice, a model in which the expression of apoE is reduced by 95% but plasma lipid levels are comparable to *Apoe^−/−^ Ldlr^−/−^* mice on a Western-type diet. These hypomorphic mice exhibit lower levels of leukocytes, including monocytes, in the blood and increased levels of HDL cholesterol [45]. The authors suggest that the secretion of apoE, including that secreted by macrophages, displaces apoA-I from VLDL, thus promoting the formation of the discoidal HDL that interacts with the ABC transporters to remove the excess cholesterol from the HSPC, resulting in the restoration of HSPC replication to normal levels. This also likely contributes to reduction in activation of monocytes and macrophages. Thus, there are several potential mechanisms by which apoE can influence monocytosis.

## 6. ApoE Mimetic Peptides and Reverse Cholesterol Transport

HDL-apolipoprotein mimetics have commanded much attention as a potential agent for the treatment of cardiovascular disease and other inflammatory diseases [46]. Many of these mimetics are based upon the amphipathic α-helical structure of the soluble apolipoproteins, particularly apoA-I. As mentioned above, the capacity of apoE to promote cholesterol efflux is embedded in its C-terminal domain, which contains amphipathic α-helical structures [18]. Based upon the recognition of this function of the C-terminal domain, amino acid residues in this region of human apoE were used as the basis for the development of apoE-derived mimetics, each containing 26 amino acids. ATI-5261, which is based on amino acids 238–266 of human apoE, was the first developed [47,48]. Although this peptide was effective in promoting cholesterol efflux in vitro and macrophage to feces reverse cholesterol transport in vivo and in reducing atherosclerosis in *Apoe^−/−^* mice, it exhibited muscle toxicity. A closely related peptide, CS-6253, has similar properties, but without the toxicity [48,49]. The precise mechanism by which these peptides attenuate atherosclerosis in vivo remains to be established, but one mechanism may be via promoting cholesterol efflux from arterial wall macrophages.

## 7. Summary

While apoA-I /HDL clearly have an important role in promoting macrophage cholesterol efflux and reverse cholesterol transport, the studies summarized in this review demonstrate that apoE also can influence cholesterol efflux and reverse cholesterol transport in particular contexts (cell culture vs in vivo) and in certain cell types. The role of cholesterol loading of macrophages in regulating apoE transcription is not fully clarified in these contexts. ApoE mimetic peptides that promote cholesterol efflux and reverse cholesterol efflux, perhaps in combination with apoE mimetic peptides that promote the clearance of apoB-containing lipoproteins [50], have the potential for reducing atherogenesis. Most of the studies reviewed have employed radiolabeled cholesterol and have not assessed the full cholesterol homeostasis in macrophages. The entrapment of macrophages of various tissue sources in alginate capsules in vivo offers an unexplored opportunity to attempt to resolve these questions, using the same macrophages in culture and in vivo. This complexity is preliminarily illustrated in a recent publication [17].

## Abbreviations

ABCA1	ATP binding cassette A1
ABCG1	ATP binding cassette G1
AcLDL	acetylated LDL
Apo	apoprotein
CETP	cholesteryl ester transfer protein
HDL	high-density lipoprotein
HSPC	hemopoietic stem cell progenitor cells
LDL	low-density lipoprotein
SR-BI	scavenger receptor B1
TICE	transintestinal cholesterol excretion
VLDL	very low density lipoprotein
